# Carotid Intima–Media Thickness Is Associated with Long-Term Mortality in Patients with Non-ST Segment Elevation Myocardial Infarction

**DOI:** 10.3390/jcm14134461

**Published:** 2025-06-23

**Authors:** Ayse Selcan Koc, Abdullah Eren Cetin, Yahya Kemal Icen, Hilmi Erdem Sumbul, Mehmet Ugurlu, Ugur Can Izlimek, Mevlut Koc

**Affiliations:** 1 Department of Radiology, University of Health Sciences—Adana Health Practice and Research Center, Adana 01230, Turkey; 2 Department of Cardiology, 25 Aralık State Hospital, Gaziantep 27060, Turkey; md.a.erencetin@gmail.com; 3 Department of Cardiology, University of Health Sciences—Adana Health Practice and Research Center, Adana 01230, Turkey; dryahyakemalicen@gmail.com (Y.K.I.); mevlutkoc78@yahoo.com (M.K.); 4 Department of Internal Medicine, University of Health Sciences—Adana Health Practice and Research Center, Adana 01230, Turkey; erdemsumbul@gmail.com; 5 Department of Cardiology, Bayindir Hospital, Istanbul 34752, Turkey; mehmetugurlu@yahoo.com; 6 Department of Internal Medicine, 5 Ocak State Hospital, Adana 01100, Turkey; ucizlimek@gmail.com

**Keywords:** NSTEMI, mortality, carotid IMT

## Abstract

**Background**: There is insufficient data in the literature on the relationship between carotid intima–media thickness (cIMT) measured in non-ST segment elevation myocardial infarction (NSTEMI) and cardiovascular (CV) mortality. Therefore, we aimed to determine the effect of cIMT value on long-term mortality in patients with NSTEMI. **Methods:** This retrospective cohort study included 279 patients with NSTEMI. In addition to clinical, demographic, laboratory, and angiographic investigations, cIMT, femoral IMT (fIMT), and aortic IMT (aIMT) were measured by B-mode ultrasonography. All patients received follow-up evaluation for CV mortality. The patients were grouped as with and without mortality. **Results**: Patients with NSTEMI received follow-up evaluations for 7.51 ± 0.85 years and 77 (27.6%) patients had mortality. Age, creatinine, blood urea nitrogen, cIMT, aIMT, fIMT, and SYNTAX score values were significantly higher in patients with mortality compared to patients without mortality. Hemoglobin, total cholesterol, LDL cholesterol, triglyceride levels, and left ventricular ejection fraction were significantly lower in patients with mortality compared to patients without mortality. In multivariate analysis, cIMT, age, and creatinine level were found to be independent predictors of mortality. Among these parameters, an increase in age (each year), carotid IMT (each 0.1 mm), and serum creatinine (each 0.1 mg/L) levels predicted an increase in mortality by 8%, 46.5%, and 12.6%, respectively. In ROC analysis, age, cIMT, and creatinine level were found to determine the development of mortality due to NSTEMI with acceptable sensitivity and specificity when an age of 65 years, 0.80 mm, and 0.90 mg/L were taken as cut-off values, respectively. **Discussion**: In patients with NSTEMI, cIMT measurement is independently associated with the development of long-term mortality.

## 1. Introduction

Acute coronary syndrome (ACS) clinically includes three different conditions: (i) unstable angina pectoris; (ii) non-ST segment elevation myocardial infarction (NSTEMI); and (iii) ST segment elevation myocardial infarction (STEMI) [1]. In the acute phase, the prognosis of STEMI patients is worse than NSTEMI patients, whereas, in the long term, the prognosis of STEMI and NSTEMI patients is similar [1,2].

Common carotid intima–media thickness (cIMT) measurement is a simple, non-invasive, and reproducible B-mode ultrasonography (US) examination. Increased cIMT has been shown to be associated with cardiovascular (CV) risk factors, coronary atherosclerosis, risk of acute myocardial infarction, and the presence of complex and extensive coronary artery disease (CAD) [3,4,5,6,7,8]. In our previous study, aortic IMT (aIMT) was shown to be an independent predictor of CAD severity in patients with NSTEMI [3]. In the same study, cIMT value was also reported to be closely associated with CAD severity [3].

Different results have been reported in several studies on the prognostic significance of cIMT measurement in ACS patients [9,10]. In a 6-month follow-up study in NSTEMI patients, it was reported that there was no close relationship between cIMT and prognosis [9]. To the best of our knowledge, no data were found in the literature on the long-term prognostic significance of the cIMT value measured in NSTEMI patients.

Therefore, we aimed to determine the effect of cIMT value on long-term CV mortality in patients with NSTEMI.

## 2. Methods

### 2.1. Study Population

This retrospective cohort study was conducted on 279 patients who were hospitalized in the coronary intensive care of our hospital between 2017 and 2018, diagnosed with NSTEMI, and received regular follow-up evaluations. The exclusion criteria were STEMI, previous percutaneous coronary intervention and coronary surgery, presence of secondary hypertension (HT), known congestive heart failure, history of cerebrovascular disease, in-hospital bleeding, severe valvular heart disease, inflammatory diseases, severe renal and hepatic diseases, hematologic diseases, presence of malignancy, pregnancy and suspected pregnancy, and age < 18 years. The necessary permissions for this study were obtained from the ethics committee of the regional hospital. All patients included in this study were informed about the study and signed an informed consent form. The follow-up period of the patients was considered as the time from the date of discharge from the hospital until the date of CV mortality.

### 2.2. Demographic, Clinical, and Laboratory Analysis

After the patients were registered as participating in this study, their medical history and physical examination data were analyzed. Demographic parameters such as age, gender, HT, diabetes mellitus (DM), hyperlipidemia, smoking, systolic blood pressure, diastolic blood pressure, and pulse rate were recorded. Complete blood count, blood urea nitrogen (BUN), creatinine, sodium, potassium, total cholesterol, low-density lipoprotein (LDL) cholesterol, high-density lipoprotein cholesterol, triglycerides, high-sensitivity troponin T, and creatine kinase–myocardial band levels measured using automated devices (Abbott Aeroset, Chiago, IL, USA) and an acceptable kit (Abbott) were noted. Left ventricular ejection fraction (LVEF) was then automatically recorded according to Simpson’s rule [11].

### 2.3. Coronary Angiography and B-Mode Ultrasound Examination

Coronary angiography was performed through femoral or radial artery access (Judkins technique). Two cardiologists evaluated the coronary angiography images individually. The SYNTAX score was calculated by including vessels with a diameter larger than 1.5 mm and stenosis over 50% from CAG images (http://www.syntaxscore.org, accessed on 11 June 2017)

**Figure 1:**
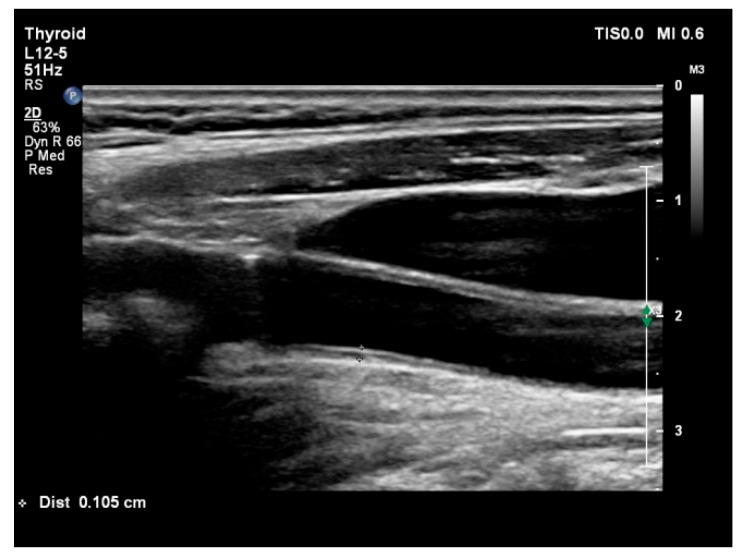
Increased common carotid intima–media thickness Doppler ultrasound image in patients with NSTEMI.

The left and right common carotid arteries, common femoral artery, abdominal aorta, and renal arteries were examined using a high-resolution ultrasound Doppler system (Philips EPIQ 7) equipped with a 12–13 MHz high-resolution linear transducer (Philips Health Care, Bothell, WA, USA). The subjects were examined in the supine position. The patients’ heads were turned 45° from the site being scanned for carotid artery screening and common cIMT was measured from the far wall of the right and left carotid artery within 10 mm proximal to bifurcation on two-dimensional ultrasound images (Figure 1). The common femoral artery was assessed and femoral IMT (fIMT) was obtained 1–2 cm proximal from the bifurcation. The aIMT was investigated in the segment of the abdominal aorta between the renal artery bifurcation and the iliac artery bifurcation. The IMT measured from the posterior wall of the abdominal artery was accepted as aIMT. All US examination time was approximately 20–30 min. The subjects were evaluated by 2 well-experienced radiology specialists for conventional and Doppler US examinations. The specialists had more than 15 years of experience in US studies and at least 1000 Doppler US procedures in a year.

### 2.4. Statistical Analysis

All analyses were performed using SPSS 23.0 (SPSS for Windows 20.0, Chicago, IL, USA). Continuous variables in group data were expressed as mean ± standard deviation. Categorical variables were expressed as numbers and percentages. The kappa coefficient was used to examine the inter–intraobserver variability of Doppler US parameters and SYNTAX score. The normality of the distribution of continuous variables was evaluated with the “Kolmogorov–Smirnov” test. In the comparison of countable parameters between the 2 groups, the Student T test and Mann–Whitney U test were used according to normal and non-normal distribution, respectively. The chi-square test was used for the comparison of categoric variables. All parameters that were significant in the univariate analysis (*p* < 0.05) were evaluated by multivariate logistic regression analysis to identify patients with CV mortality. Receiver operating characteristic (ROC) curve analysis was performed to determine the cut-off value of parameters that were independently predictive of CV mortality. Statistical significance was defined as a *p* value < 0.05 for all comparisons.

## 3. Results

In this study, 279 patients (146 males, 133 females, age 63.7 ± 12.4 years) who were receiving long-term follow-up evaluation for NSTEMI were evaluated. The follow-up period was 7.51 ± 0.85 years and 77 (27.6%) patients had CV mortality. The patients were grouped as with and without CV mortality. The parameters determining CV mortality were evaluated. The Cohen kappa values that evaluate the inter–intraobserver variability were over 90% for all Doppler US parameters and SYNTAX score values (*p* < 0.001 for all comparisons).

### 3.1. Demographic and Clinical Data of Patients with and Without Cardiovascular Mortality

Demographic and clinical data of patients with and without mortality are shown in Table 1. Age was found to be significantly higher in patients with mortality compared to patients without mortality. Other demographic and clinical data were similar between the two groups (Table 1).

### 3.2. Laboratory Data of Patients with and Without Cardiovascular Mortality

Laboratory data of patients with and without mortality are shown in Table 2. Among these parameters, creatinine, BUN, and Hs troponin T serum levels were significantly higher in patients with mortality compared to those without mortality. Hemoglobin, total cholesterol, LDL cholesterol, and triglyceride serum levels were significantly lower in patients with mortality compared to those without mortality. Other laboratory data were similar between the two groups (Table 2).

### 3.3. Angiographic, Echocardiographic, and Ultrasound Findings in Patients with and Without Cardiovascular Mortality

Angiographic, echocardiographic, and US findings of patients with and without mortality are shown in Table 3. Among these parameters, cIMT, aIMT, fIMT, and SYNTAX score values were significantly higher in patients with mortality compared to those without mortality. LVEF was found to be significantly lower in patients with mortality compared to those without mortality. Other laboratory data were similar between the two groups (Table 3).

### 3.4. Identification of Parameters That Independently Identify Patients with Cardiovascular Mortality

**Figure 2:**
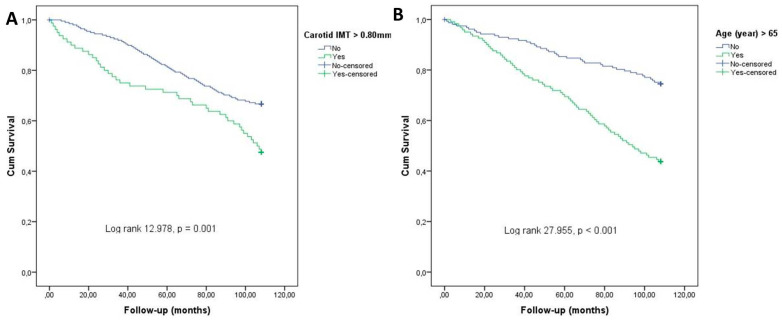
Kaplan–Meier survival curves for NSTEMI patients with long-term mortality. (**A**) Presence or absence of cIMT > 0.80 mm; (**B**) presence or absence of age > 65 years.

Multivariate logistic regression analysis was performed to determine the parameters that were closely and independently associated with mortality among all parameters associated with the development of mortality in the patients included in this study. As a result of this analysis, age, cIMT, and creatinine level were found to independently determine patients with mortality (Table 4). Among these parameters, an increase in age (each year), carotid IMT (each 0.1 mm), and serum creatinine (each 0.1 mg/L) levels predicted an increase in mortality by 8%, 46.5%, and 12.6%, respectively. LVEF, SYNTAX score, and hs-TnT levels, which were associated with mortality in univariate analysis, were not found to be independent predictors of mortality in patients with NSTEMI (Table 4). The Kaplan–Meier survival curves in Figure 2 show that the presence of cIMT > 0.8 mm and age > 65 years were significantly related to CV mortality in patients with NSTEMI.

### 3.5. ROC Curve Analysis for Parameters That Independently Identify Patients with Cardiovascular Mortality

**Figure 3:**
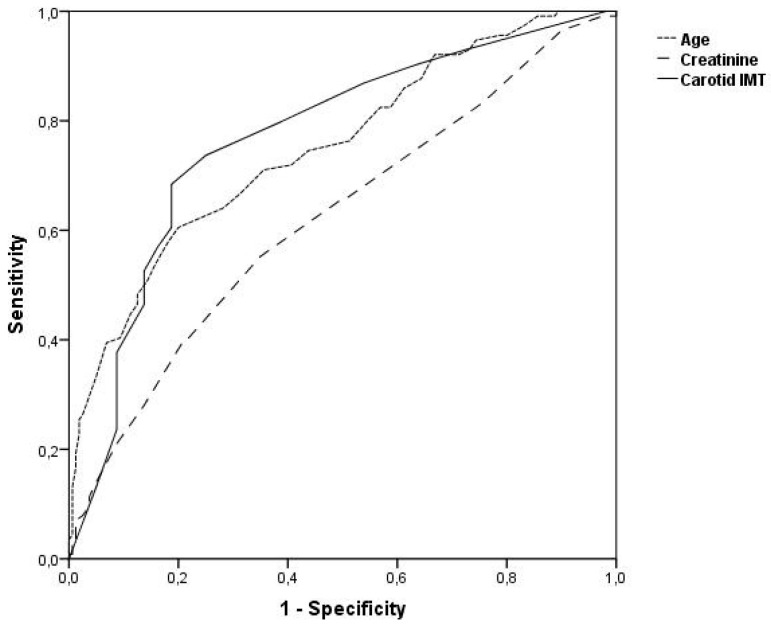
ROC curve analysis of age, cIMT, and creatinine level as predictors of CV mortality.

When ROC curve analysis was performed for age, cIMT, and creatinine level in terms of determining mortality, it was found that all three parameters determined mortality independently and the area under the ROC curve was 0.751, 0.769, and 0.622 for age, cIMT, and creatinine level, respectively (Figure 3, Table 5). Age, cIMT, and creatinine level were found to determine the development of mortality due to NSTEMI with acceptable sensitivity and specificity when 65 years, 0.80 mm, and 0.90 mg/L were taken as cut-off values, respectively (Table 5).

## 4. Discussion

The main findings of this study can be summarized as follows: (1) cIMT measurement is independently associated with the development of long-term mortality in patients with NSTEMI and this finding has been shown for the first time in this study; (2) age and creatinine level measured at admission are the other parameters independently associated with mortality in patients with NSTEMI and this data is consistent with previous studies in the literature; and (3) cIMT was found to determine the development of mortality due to NSTEMI with acceptable sensitivity and specificity when 0.80 mm was taken as the cut-off value.

When long-term (>10 years) mortality analysis was evaluated in NSTEMI and STEMI patients, the mortality rates of both diseases were reported to be similar and approximately 21% [12]. In our study, long-term mortality was also evaluated and unlike the previous study, the mortality rate was 27.6%. The reason why the mortality rate in our study is higher than the previous study is thought to be due to the fact that the patients included in this study were more elderly.

TIMI risk score and GRACE risk score are used to predict short-term and long-term mortality in patients with NSTEMI [1]. TIMI risk score is a short-term (<14 days) prognosis predictor and is generally used to determine the patients who will undergo early intervention in NSTEMI cases [1]. The GRACE risk score is used as a long-term (6 months, 1 year, and 3 years) prognosis predictor except for in-hospital short-term prognosis [1]. The GRACE risk score includes age, Killip classification, systolic blood pressure, heart rate, ST segment deviation, presence of cardiac arrest on admission, serum creatinine level, and increased cardiac biomarkers [1]. In our study, among these parameters, age and serum creatinine level were found to be independent predictors of very long-term mortality.

Advanced age is the most important parameter found together in all risk score systems used in both STEMI and NSTEMI cases. Especially in NSTEMI cases, ≥65 years of age is given as a cut-off value. In our study, advanced age was determined to be an important and independent parameter for mortality and the best sensitivity and specificity were obtained when ≥65 years of age was taken as the cut-off value for mortality determination. Our study also showed that high Hs troponin T and LVEF values, which were also found in previous scores, were associated with prognosis, although not independently.

However, the cIMT value, which was absent in both the GRACE and TIMI risk scores, was found to be an independent predictor of long-term mortality. cIMT measurement has been shown to be a prognostic marker that predicts the development of CV events in patient groups with advanced age [13], HT [14], stable CAD [15], STEMI [10], DM [16], and no known vascular disease [17]. In a study by Lee S. et al. [10], cIMT was shown to predict the development of future CV events in 345 patients with STEMI. Tello-Montoliu A. et al. [9] reported that cIMT value was not associated with CV event development in 126 NSTEMI patients. In the same study, it was emphasized that cIMT value was independently associated with age and the presence of DM. However, in a study investigating the prognostic significance of cIMT, 126 patients were evaluated, and the patients received follow-up evaluation for only 6 months [9]. In our study, unlike the previous study, 279 NSTEMI cases were included, and the mean follow-up period was 7.5 years. In our study, unlike previous studies, IMT measurements were made from three different anatomical regions, and it was shown that cIMT measurement had more prognostic significance than fIMT and aIMT measurements. Previously, including the study conducted by us, it has been shown that cIMT increase is associated with CAD complexity and severity [3,7]. We thought that the reason why cIMT is a long-term prognostic indicator may be related to this situation.

Carotid Doppler ultrasonography (US) can be performed easily, safely, and accurately by a skilled radiologist. Carotid intima–media thickness (cIMT) measurements obtained through this examination may increase particularly in the early or subclinical phases of the atherosclerotic process. In clinical practice, a cIMT value > 0.9 mm is generally considered indicative of increased cIMT or subclinical atherosclerosis [18]. Moreover, a cIMT value ≥ 1.5 mm is accepted as representing a carotid plaque and is thought to be a more meaningful marker of atherosclerotic burden than cIMT alone [19].

Although no well-defined cIMT cut-off value has been established for prognostic assessment in NSTEMI patients, a study involving approximately 2 years of follow-up evaluations in STEMI patients reported that both a cIMT value ≥ 0.83 mm and the presence of carotid plaque were significantly associated with increased mortality [10]. In our study, we demonstrated that a cIMT value > 0.80 mm independently predicted long-term mortality in patients with NSTEMI. This threshold may be considered a practical and applicable marker for use in daily clinical practice.

## 5. Limitations

This study has several important limitations. This study was retrospective and conducted at a single center. cIMT ≥ 1.5 mm is considered as carotid plaque. In several studies, cIMT and the presence of carotid plaque were evaluated together in predicting CV events and mortality, and it was shown that the presence of carotid plaque was a better predictor of CV events and mortality and had better prognostic power than cIMT [15,20,21]. Following these findings, it has been suggested that carotid plaque may be used as a secondary prevention and prognosis factor in CAD patients. However, the prognostic significance of cIMT and carotid plaque was not compared in our study because of the small number of patients with carotid plaque. Since GRACE and TIMI risk scores were not performed in our previous study [3], these scores were not used in this study. If these two risk scores had been evaluated in the previous study, the prognostic significance of cIMT might have been more significant. Another important cause of mortality in patients with acute coronary syndrome (ACS) is bleeding, both in-hospital and post-discharge. A recently published study demonstrated that in-hospital bleeding independently predicted 1-year mortality in patients hospitalized with ACS [22]. In our study, we did not evaluate the association between bleeding and mortality, as patients with in-hospital bleeding were excluded based on our study design.

Other prognostic factors in NSTEMI patients include medical therapies (e.g., statins, antiplatelet agents, and beta-blockers), Killip classification, and prior cardiovascular history. Unfortunately, these parameters were not included in the analysis of our study [1,2].

Recent evidence also suggests that anti-diabetic agents such as sodium–glucose co-transporter-2 (SGLT-2) inhibitors and glucagon-like peptide-1 (GLP-1) receptor agonists may have cardioprotective effects in ACS patients beyond their glycemic benefits [22]. However, the potential short- and long-term benefits of these agents were not evaluated in our study.

The inclusion and analysis of all these parameters—GRACE and TIMI risk scores, in-hospital bleeding, medical therapy, Killip class, history of prior cardiovascular disease, and use of SGLT-2 inhibitors and GLP-1 receptor agonists—would likely have provided more comprehensive and meaningful prognostic insights.

## 6. Conclusions

In conclusion, the cIMT value measured with B-mode US in NSTEMI patients may be a useful parameter in determining long-term mortality in these patients. cIMT measurement and follow-up evaluation are not conducted in the hospitalization and follow-up care of both NSTEMI and STEMI patients. In addition to routine clinical, demographic, and laboratory parameters, cIMT measurement, which is a simple, inexpensive, reproducible, and objective parameter, can be added as a routine examination in NSTEMI patients. However, prospective, randomized, and multicenter studies are required.

## Figures and Tables

**Table 1:** Demographic and clinical data of NSTEMI patients with and without cardiovascular mortality.

Variables	Mortality (+) n = 77	Mortality (−) n = 202	*p*
Age (year)	59.1 ± 10.7	70.3 ± 11.5	**<0.001**
Gender (female/male), n	38/39	95/107	0.337
Hypertension, n (%)	44 (57%)	105 (52%)	0.260
Diabetes mellitus, n (%)	42 (54%)	103 (51%)	0.895
Smoking, n (%)	35 (45%)	89 (44%)	0.809
Hypercholesterolemia, n (%)	40 (48%)	101 (50%)	0.808
Systolic blood pressure (mmHg)	117 ± 17	111 ± 16	0.502
Diastolic blood pressure (mmHg)	76 ± 7.8	77 ± 7.7	0.302
Heart rate (beats/minute)	84 ± 16	83 ± 17	0.770

Statistically significant *p* values are shown in bold.

**Table 2:** Laboratory data of NSTEMI patients with and without cardiovascular mortality.

Variables	Mortality (+) n = 77	Mortality (−) n = 202	*p*
White blood cell (10^3^/µL)	10.1 ± 3.7	9.5 ± 3.2	0.209
Hemoglobin (g/dL)	12.8 ± 2.9	13.5 ± 1.7	**0.023**
Creatinine (mg/dL)	1.11 ± 0.98	0.85 ± 0.51	**0.012**
Blood urea nitrogen (mg/dL)	42.7 ± 21.8	32.1 ± 11.7	**0.001**
Sodium (mmol/L)	139 ± 3.9	137 ± 4.9	0.182
Potassium (mmol/L)	4.44 ± 0.52	4.34 ± 0.47	0.085
Total cholesterol (mg/dL)	167 ± 46	189 ± 49	**0.001**
Low-density lipoprotein cholesterol (mg/dL)	102 ± 32	112 ± 43	**0.033**
High-density lipoprotein cholesterol (mg/dL)	41 ± 13	41 ± 11	0.923
Triglycerides (mg/dL)	149 ± 101	183 ± 89	**0.032**
Hs troponin T level (ng/mL)	993 ± 1946	447 ± 1246	**0.024**
Creatine kinase–myocardial band (U/L)	17.2 ± 35.2	15.3 ± 38.3	0.703

Statistically significant *p* values are shown in bold.

**Table 3:** Angiographic, echocardiographic, and ultrasound findings in NSTEMI patients with and without cardiovascular mortality.

Variables	Mortality (+) n = 77	Mortality (−) n = 202	*p*
LVEF (%)	51.7 ± 8.7	54.1 ± 7.8	**0.026**
Carotid IMT (mm)	0.81 ± 0.21	0.71 ± 0.17	**<0.001**
Aortic IMT (mm)	1.69 ± 0.84	1.29 ± 0.71	**<0.001**
Femoral IMT (mm)	0.96 ± 0.29	0.81 ± 0.25	**<0.001**
SYNTAX score	17.3 ± 9.8	12.3 ± 8.5	**<0.001**

LVEF: Left ventricular ejection fraction, IMT: intima–media thickness. Statistically significant *p* values are shown in bold.

**Table 4:** Multivariate logistic regression analysis to identify NSTEMI patients with cardiovascular mortality.

	Odds	95% CI	*p*
Carotid IMT (each 0.1 mm)	1.465	1.224–1.750	**<0.001**
Age (each year)	1.080	1.051–1.110	**0.001**
Creatinine (each 0.1 mg/L)	1.126	1.034–1.226	**0.008**
LVEF (%)	0.982	0.949–1.017	0.308
SYNTAX score	1.012	0.917–1.081	0.235
Hs troponin T level (each 10 ng/mL)	1.015	0.996–1.034	0.126

LVEF: Left ventricular ejection fraction, IMT: intima–media thickness. Statistically significant *p* values are shown in bold.

**Table 5:** ROC curve analysis for identifying NSTEMI patients with cardiovascular mortality.

Variable	AUROC Curve	*p*	Cut-Off	Sensitivity	Specificity
Carotid IMT	0.769 (0.712–0.827)	**<0.001**	0.80 mm	79.8%	75.1%
Age	0.751 (0.693–0.810)	**<0.001**	65 year	72.0%	71.3%
Creatinine	0.622 (0.555–0.690)	**0.001**	0.90 mg/L	65.8%	65.1%

IMT: Intima–media thickness. Statistically significant *p* values are shown in bold.

## Data Availability

The data presented in this study are available upon request from the corresponding author.

## References

[1] Rao S.V., O’Donoghue M.L., Ruel M., Rab T., Tamis-Holland J.E., Alexander J.H., Baber U., Baker H., Cohen M.G., Cruz-Ruiz M. (2025). 2025 ACC/AHA/ACEP/NAEMSP/SCAI Guideline for the Management of Patients with Acute Coronary Syndromes: A Report of the American College of Cardiology/American Heart Association Joint Committee on Clinical Practice Guidelines. Circulation.

[2] Bouisset F., Ruidavets J.B., Dallongeville J., Moitry M., Montaye M., Biasch K., Ferrières J. (2021). Comparison of Short- and Long-Term Prognosis between ST-Elevation and Non-ST-Elevation Myocardial Infarction. J. Clin. Med..

[3] Icen Y.K., Koc A.S., Sumbul H.E. (2019). Coronary Artery Disease Severity Is Associated with Abdominal Aortic Intima-Media Thickness in Patients with Non-ST-Segment Elevation Myocardial Infarction. Angiology.

[4] Simon A., Gariepy J., Chironi G., Megnien J.L., Levenson J. (2002). Intima-media thickness: A new tool for diagnosis and treatment of cardiovascular risk. J. Hypertens..

[5] Burke G.L., Evans G.W., Riley W.A., Sharrett A.R., Howard G., Barnes R.W., Rosamond W., Crow R.S., Rautaharju P.M., Heiss G. (1995). Arterial wall thickness is associated with prevalent cardiovascular disease in middle-aged adults: The Atherosclerosis Risk in Communities (ARIC) Study. Stroke.

[6] Bots M.L., Grobbee D.E., Hofman A., Witteman J.C. (2005). Common carotid intima-media thickness and risk of acute myocardial infarction: The role of lumen diameter. Stroke.

[7] Kato M., Dote K., Habara S., Takemoto H., Goto K., Nakaoka K. (2003). Clinical implications of carotid artery remodeling in acute coronary syndrome: Ultrasonographic assessment of positive remodeling. J. Am. Coll. Cardiol..

[8] Demircan S., Tekin A., Tekin G., Topçu S., Yiğit F., Erol T., Katircibaşi T., Sezgin A.T., Baltali M., Ozin B. (2005). Comparison of carotid intima-media thickness in patients with stable angina pectoris versus patients with acute coronary syndrome. Am. J. Cardiol..

[9] Tello-Montoliu A., Moltó J.M., López-Hernández N., García-Medina A., Roldán V., Sogorb F., Lip G.Y., Marín F. (2007). Common carotid artery intima-media thickness and intracranial pulsatility index in non-ST-elevation acute coronary syndromes. Cerebrovasc. Dis..

[10] Lee S., Cho G.Y., Kim H.S., Yoon Y.E., Lee S.P., Kim H.K., Kim Y.J., Sohn D.W. (2014). Common carotid intima-media thickness as a risk factor for outcomes in Asian patients with acute ST-elevation myocardial infarction. Can. J. Cardiol..

[11] Lang R.M., Bierig M., Devereux R.B., Flachskampf F.A., Foster E., Pellikka P.A., Picard M.H., Roman M.J., Seward J., Shanewise J.S. (2005). Recommendations for chamber quantification: A report from the American Society of Echocardiography’s Guidelines and Standards Committee and the Chamber Quantification Writing Group, developed in conjunction with the European Association of Echocardiography, a branch of the European Society of Cardiology. J. Am. Soc. Echocardiogr..

[12] Lorenz M.W., Schaefer C., Steinmetz H., Sitzer M. (2010). Is carotid intima media thickness useful for individual prediction of cardiovascular risk? Ten-year results from the Carotid Atherosclerosis Progression Study (CAPS). Eur. Heart J..

[13] Murakami S., Otsuka K., Hotta N., Yamanaka G., Kubo Y., Matsuoka O., Yamanaka T., Shinagawa M., Nunoda S., Nishimura Y. (2005). Common carotid intima-media thickness is predictive of all-cause and cardiovascular mortality in elderly community-dwelling people: Longitudinal Investigation for the Longevity and Aging in Hokkaido County (LILAC) study. Biomed. Pharmacother..

[14] Zielinski T., Dzielinska Z., Januszewicz A., Rynkun D., Makowiecka Ciesla M., Tyczynski P., Prejbisz A., Demkow M., Kadziela J., Naruszewicz M. (2007). Carotid intima-media thickness as a marker of cardiovascular risk in hypertensive patients with coronary artery disease. Am. J. Hypertens..

[15] Keo H.H., Baumgartner I., Hirsch A.T., Duval S., Steg P.G., Pasquet B., Bhatt D.L., Roether J., REACH Registry Investigators (2011). Carotid plaque and intima-media thickness and the incidence of ischemic events in patients with atherosclerotic vascular disease. Vasc. Med..

[16] Katakami N., Yamasaki Y., Kosugi K., Waki H., Matsuhisa M., Kajimoto Y., Masuyama T., Hori M. (2004). Tissue characterization identifies subjects with high risk of cardiovascular diseases. Diabetes Res. Clin. Pract..

[17] Dijk J.M., van der Graaf Y., Bots M.L., Grobbee D.E., Algra A. (2006). Carotid intima-media thickness and the risk of new vascular events in patients with manifest atherosclerotic disease: The SMART study. Eur. Heart J..

[18] Inaba Y., Chen J.A., BMazzolai L., Teixido-Tura G., Lanzi S., Boc V., Bossone E., Brodmann M., Bura-Rivière A., De Backer J. (2024). 2024 ESC Guidelines for the management of peripheral arterial and aortic diseases. Eur. Heart J..

[19] Touboul P.J., Hennerici M.G., Meairs S., Adams H., Amarenco P., Bornstein N., Csiba L., Desvarieux M., Ebrahim S., Hernandez Hernandez R. (2012). Mannheim carotid intima-media thickness and plaque consensus (2004–2006–2011). An update on behalf of the advisory board of the 3rd, 4th and 5th watching the risk symposia, at the 13th, 15th and 20th European Stroke Conferences, Mannheim, Germany, 2004, Brussels, Belgium, 2006, and Hamburg, Germany, 2011. Cerebrovasc. Dis..

[20] Yuk H.B., Park H.W., Jung I.J., Kim W.H., Kim K.H., Kim K.H., Yang D.J., Park Y.H., Kim Y.K., Song I.G. (2015). Analysis of Carotid Ultrasound Findings on Cardiovascular Events in Patients with Coronary Artery Disease during Seven-Year Follow-Up. Korean Circ. J..

[21] Park H.W., Kim W.H., Kim K.H., Yang D.J., Kim J.H., Song I.G., Kwon T.G., Bae J.H. (2013). Carotid plaque is associated with increased cardiac mortality in patients with coronary artery disease. Int. J. Cardiol..

[22] Karakasis P., Patoulias D., Kassimis G., Koufakis T., Klisic A., Doumas M., Fragakis N., Rizzo M. (2024). Therapeutic Potential of Sodium-glucose Co-transporter-2 Inhibitors and Glucagon-like Peptide-1 Receptor Agonists for Patients with Acute Coronary Syndrome: A Review of Clinical Evidence. Curr. Pharm. Des..

